# Wdpcp regulates cellular proliferation and differentiation in the developing limb via hedgehog signaling

**DOI:** 10.1186/s12861-021-00241-9

**Published:** 2021-07-05

**Authors:** Mark T. Langhans, Jingtao Gao, Ying Tang, Bing Wang, Peter Alexander, Rocky S. Tuan

**Affiliations:** 1 Department of Orthopaedic Surgery, Center for Cellular and Molecular Engineering, University of Pittsburgh School of Medicine, 450 Technology Drive, Pittsburgh, PA 15219-3143 USA; 2 Present Address: Institute for Tissue Engineering and Regenerative Medicine, The Chinese University of Hong Kong, Shatin, Hong Kong SAR, China

**Keywords:** Hedgehog, Growth plate, Limb bud, Wdpcp, Osteogenesis, Chondrogenesis, Proliferation, Differentiation

## Abstract

**Background:**

Mice with a loss of function mutation in *Wdpcp* were described previously to display severe birth defects in the developing heart, neural tube, and limb buds. Further characterization of the skeletal phenotype of *Wdpcp* null mice was limited by perinatal lethality.

**Results:**

We utilized Prx1-Cre mice to generate limb bud mesenchyme specific deletion of Wdpcp. These mice recapitulated the appendicular skeletal phenotype of the Wdpcp null mice including polydactyl and limb bud signaling defects. Examination of late stages of limb development demonstrated decreased size of cartilage anlagen, delayed calcification, and abnormal growth plates. Utilizing in vitro assays, we demonstrated that loss of Wdpcp in skeletal progenitors lead to loss of hedgehog signaling responsiveness and associated proliferative response. In vitro chondrogenesis assays showed this loss of hedgehog and proliferative response was associated with decreased expression of early chondrogenic marker N-Cadherin. E14.5 forelimbs demonstrated delayed ossification and expression of osteoblast markers Runx2 and Sp7. P0 growth plates demonstrated loss of hedgehog signaling markers and expansion of the hypertrophic zones of the growth plate. In vitro osteogenesis assays demonstrated decreased osteogenic differentiation of Wdpcp null mesenchymal progenitors in response to hedgehog stimulation.

**Conclusions:**

These findings demonstrate how Wdpcp and associated regulation of the hedgehog signaling pathway plays an important role at multiple stages of skeletal development. Wdpcp is necessary for positive regulation of hedgehog signaling and associated proliferation is key to the initiation of chondrogenesis. At later stages, Wdpcp facilitates the robust hedgehog response necessary for chondrocyte hypertrophy and osteogenic differentiation.

**Supplementary Information:**

The online version contains supplementary material available at 10.1186/s12861-021-00241-9.

## Background

The primary cilium is a transiently formed microtubule-based organelle that coordinates many signaling processes within vertebrate development and has been found in almost all vertebrate cell types [1]. There are several hundred proteins and associated complexes controlling formation and maintenance of the cilium [2]. Mutations in the genes encoding these proteins are associated with diseases known as ciliopathies with a wide array of associated phenotypes affecting almost all organ systems [3].

One the key complexes necessary for initiation of ciliogenesis is the CPLANE complex, whose members include Wdpcp, Intu, and Fuz [4]. The CPLANE complex has been shown to regulate intraflagellar transport proteins (IFT) necessary for formation of the primary cilium [5]. Several studies of the IFT proteins have demonstrated important roles for these proteins in the limb bud, growth plate [6, 7], chondrocyte differentiation [8, 9], osteoblast differentiation [10–12], and homeostasis of adult skeletal tissues [9, 13–16]. Although the role of CPLANE proteins in skeletal development has been less intensively studied, *Wdpcp*^*−/−*^ [17], *Fuz*^*−/−*^ [18], and *Intu*^*−/−*^ [19, 20] mice have been characterized. While examination of later stages in skeletal development of these mutants has been limited by lethality, analysis of the limb buds demonstrates polydactyly and altered hedgehog signaling.

An unbiased *N-*ethyl-*N-*nitrosurea (ENU) induced mutagenesis screen in mice for homozygous recessive mutations recovered the *Cys40* mouse bearing a null mutation in *Wdpcp* [17]. *Wdpcp* was first identified in *Drosophila melanogaster* as *Fritz* [21], and the vertebrate homolog was later demonstrated to play a role in hedgehog signaling and ciliogenesis [17, 22]. Mutations in *WDPCP* in humans are associated with Bardet-Biedl Syndrome and Meckel Gruber Syndrome, ciliopathy disorders with features that include polydactyly, congenital heart defects, renal abnormalities, blindness, truncal obesity, and genitourinary malformations [23].

The *Cys40* mice had disrupted ciliogenesis and displayed a gross appendicular skeletal phenotype of polydactyly and shortened, dysmorphic long bones. Analysis of the limb buds showed disrupted formation of truncated Gli3 repressor and altered hedgehog signaling. In addition to the skeletal phenotype, the *Cys40* mutant had a number of developmental defects including congenital heart malformations, cystic kidneys, tracheoesophageal fistula, and cloacal septation defects that lead to embryonic lethality. A conditional deletion mouse model of *Wdpcp* bearing LoxP sites on either side of exon 5 was confirmed to recapitulate the phenotype of the *Cys40* mutant when crossed with a constitutively expressed CMV-Cre [17].

We generated *Prx1-Cre;Wdpcp*^*Flox/Cys40*^ mice to bypass the high perinatal lethality of the *Wdpcp* constituitive knockout mice and facilitate study of the role of *Wdpcp* in later stages of skeletal development including the organization of the growth plate and ossification of appendicular skeletal elements. Histomorphometric characterization of the developing limbs demonstrated diminished size of the skeletal elements and delayed ossification. These findings corresponded with delayed expression of chondrocyte hypertrophy/early osteoblast markers. Consistent with prior studies of genes necessary for ciliogenesis [6, 12, 13, 24–26], we noted alteration of hedgehog pathway activation in the developing limb bud as well as the growth plate. These findings were explored further with in vitro assays that demonstrated decreased proliferation, and delayed early chondrogenic and osteogenic differentiation of mesenchymal progenitor cells lacking Wdpcp in response to hedgehog stimulation. In summary, we demonstrate a critical role for Wdpcp in modulating hedgehog signaling to facilitate multiple processes in mammalian appendicular skeletal development including proliferation, growth plate organization, and differentiation of skeletal precursors.

## Results

### Wdpcp is expressed in skeletal tissues, and limb mesenchyme specific deletion of Wdpcp recapitulates the Cys40 appendicular skeleton phenotype

**Fig. 1:**
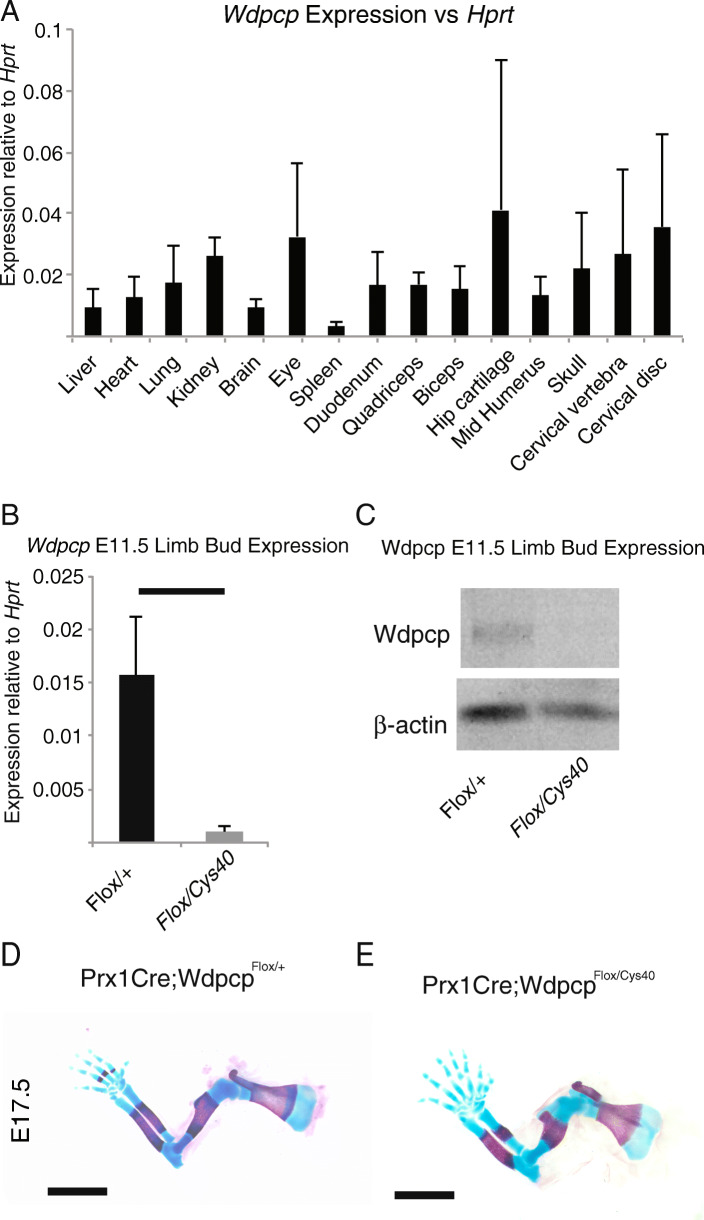
*Wdpcp* is highly expressed in musculoskeletal tissues and loss of expression in limb bud mesenchyme is associated with ossification, patterning, and growth defects. **A** RT-PCR analysis of *Wdpcp* expression in several adult tissues. **B** Expression of *Wdpcp* RNA transcripts and (**C**) Wdpcp protein expression in E11.5 limb buds from *Prx1-Cre;Wdpcp*^*Flox/+*^ (control) and *Prx1-Cre;Wdpcp*^*Flox/Cys40*^ (Wdpcp-cKO) mice. **D**, **E** E17.5 forelimbs stained with Alizarin Red and Alcian Blue showing areas of ossification (red) and cartilaginous condensation (blue) as well as gross skeletal phenotype of *Prx1-Cre;Wdpcp*^*Flox/+*^ (control) and *Prx1-Cre;Wdpcp*^*Flox/Cys40*^ (Wdpcp-cKO) mice. Black scale bars represent 2 mm in all panels

Quantification of RNA transcripts of *Wdpcp* in multiple tissues confirmed that *Wdpcp* is widely expressed and is enriched in musculoskeletal tissues (Fig. 1A). We chose to focus our studies specifically on the appendicular skeleton and wanted to determine the spatiotemporal requirement for *Wdpcp* for normal appendicular skeletogenesis. Previous conditional gene knockout studies in mice had demonstrated proteins necessary for normal primary cilia formation functioned within the limb bud mesenchyme to facilitate skeletogenesis [12]. Based on these studies, we hypothesized that *Wdpcp* deletion from the limb bud mesenchyme would recapitulate the *Cys40* phenotype. We utilized a conditional allele of *Wdpcp* in combination with the *Prx1-Cre* mouse to confirm that *Wdpcp* functions within the limb bud mesenchyme. The *Prx1-Cre* mouse expresses Cre recombinase in the forelimb bud mesenchyme at E9.5 and in the hindlimb bud mesenchyme at E10.5 [27]. Mice with a limb bud mesenchyme specific deletion of *Wdpcp* were generated by crossing the *Prx1-Cre;Wdpcp*^*Cys40/+*^ mice to the *Wdpcp*^*Flox/Flox*^ mice. Quantification of RNA transcripts as well as Western Blot for Wdpcp protein confirmed deletion of Wdpcp in E11.5 limb buds (Fig. 1B,C). Alizarin Red/Alcian Blue staining of E17.5 *Prx1-Cre;Wdpcp*^*Flox/+*^ mice (control) (Fig. 1D) and *Prx1-Cre;Wdpcp*^*Flox/Cys40*^ (Wdpcp-cKO) mice (Fig. 1E) revealed a similar phenotype to that observed in the *Cys40* mutant including severe polydactyly with shortened, dysmorphic long bones [17].

### Prx1-Cre;Wdpcp^Flox/Cys40^ mice have smaller cartilage anlagen, delayed ossification associated with delayed expression of chondrocyte hypertrophic marker, and expanded zone of hypertrophy in growth plates

**Fig. 2:**
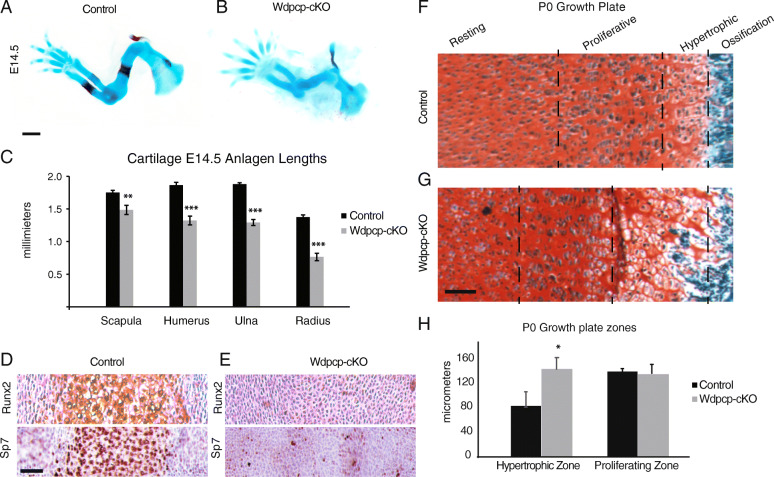
Developing limbs of Wdpcp-cKO embryos have smaller appendicular skeletal cartilage anlagen, delayed expression of hypertrophic chondrocyte markers and expanded hypertrophic zones in growth plate. **A**-**C** Alcian Blue and Alizarin Red staining of E14.5 embryos with quantification of anlagen lengths. **D**, **E** Immunohistochemistry staining of E14.5 humerus for chondrocyte hypertrophy markers Runx2 and Sp7. **F**, **G** Safranin O Fast Green staining of P0 growth plates and (**H**) quantification of growth plate zone sizes. Scale bars are 1 mm for (**A**, **B**), and 50 μm for (**D**-**G**). Error bars represent one standard deviation. Wdpcp-cKO is compared to matched control with * for *p* < 0.05, ** for *p* < 0.01, and *** for *p* < 0.001

Histomorphometric characterization of E14.5 fore limb buds was notable for significantly decreased size of cartilage anlagen in *Prx1-Cre;Wdpcp*^*Flox/Cys40*^ (referred to as Wdpcp-cKO) mice versus *Prx1-Cre;Wdpcp*^*Flox/+*^ (referred to as control) mice. There was a trend toward progressive shortening with more distal elements (Fig. 2A-C). Additionally, it was noted that at E14.5 there was delayed ossification in the Wdpcp-cKO mice as indicated by lack of Alizarin Red staining (Fig. 2A-B). To explore this finding further, we performed immunohistochemical staining for early chondrocyte hypertrophy markers Runx2 (Cbfa1) and Sp7 (Osterix) expression of which immediately precedes calcification and vascular invasion [28]. While the controls demonstrated expected staining at the humerus primary center of ossification, there was no significant staining in the Wdpcp-cKO humerus (Fig. 2D, E). The P0 growth plates of the Wdpcp-cKO mice demonstrated an expanded zone of hypertrophic chondrocytes with loss of the clear zonal demarcation of the prehypertrophic and hypertrophic zones (Fig. 2F-H).

### Hedgehog signaling is disrupted at multiple stages of skeletal development in Wdpcp-cKO mice

Given that prior studies of Wdpcp reported hedgehog signaling dysfunction in the limb bud [17] and the central role this pathway plays in several aspects of skeletal development including chondrogenesis, osteogenesis, and growth plate patterning [29], we chose to investigate the effects of Wdpcp-cKO on hedgehog signaling. We utilized Gli1 expression, shown by the LacZ reporter, as marker of hedgehog pathway activity [30].

**Fig. 3:**
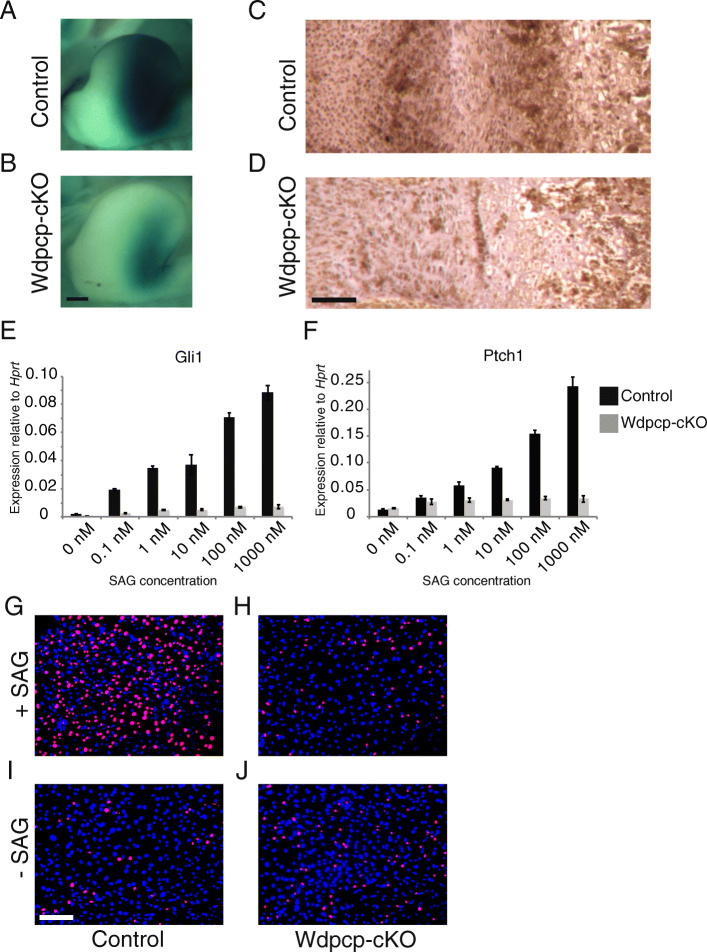
Wdpcp-cKO embryos have disrupted hedgehog signaling in developing limb bud and growth plate, and Wdpcp-cKO mesenchymal progenitors show diminished response to hedgehog stimulation. **A**, **B** Gli1-Lacz reporter mice were used to observe hedgehog signaling activity (blue) in Wdpcp-cKO versus control E11.5 limb buds. **C**, **D** Immunohistochemistry of P0 growth plates for hedgehog signaling reporter Gli1 in Wdpcp-cKO versus control. **E**, **F** Expression of hedgehog reporter genes *Gli1* and *Ptch1* in mesenchymal progenitor cells from Wdpcp-cKO embryos with increasing dosage of smoothened agonist (SAG). Error bars represent one standard deviation. **G**-**J** EdU staining for proliferative cells (red) in mesenchymal progenitor cells from Wdpcp-cKO and control with and without SAG treatment. Scale bars represent 200 μm in (**A**, **B**) and 50 μm in (**C**, **D**); white scale bar represents 50 μm in G-J

*Gli1-LacZ;Prx1-Cre;Wdpcp*^*Flox/Cys40*^ (Wdpcp-cKO) E11.5 limb buds demonstrated significantly diminished staining versus control consistent with lack of full activation of the hedgehog pathway (Fig. 3A, B). Similarly, while Gli1 expression in P0 growth plate is typically enriched in the prehypertrophic and early proliferating chondrocytes (Fig. 3C), the Wdpcp-cKO growth plates demonstrated a distinct lack of staining or patterning (Fig. 3D). Previously published data showed failure of Gli3 repressor processing in the E10.5 limb bud [17]. This would theoretically lead to a lack of Gli3 repressor and abnormal activation of hedgehog pathway in contrast to what we observed at E11.5. The discordance of these results highlights the importance of the Wdpcp in not only repression, but also full activation of the hedgehog pathway. The importance of finely tuned hedgehog pathway sensitivity to limb development has been highlighted by recent studies [31]. Our findings suggest that in the developing limb Wdpcp is necessary not only for full repression of hedgehog signaling but also for full activation. To investigate this further, we examined the responsiveness of limb mesenchymal progenitor cells to Smo stimulation with SAG treatment, and saw a significantly diminished responsiveness in the Wdpcp-cKO cells in expression of the two hedgehog pathway responsive genes *Gli1* and *Ptch1* [32] (Fig. 3E, F) that correlated with decreased cilia formation (supplementary Fig. 2). Several reports have suggested that one of the primary functions of hedgehog signaling in mesenchymal progenitors is control of proliferation necessary to achieve cell density required for differentiation of skeletal tissue [33–35]. We examined proliferation of mesenchymal progenitors in response to Smo agonist (SAG) and saw a dramatic response in control cells (Fig. 3G, I) versus a lack of proliferative response in Wdpcp-cKO cells (Fig. 3H, J). These results are in line with the histologically observable loss of clear demarcation of the proliferative zone in Wdpcp-cKO growth plates as well as the smaller anlagen size.

### Loss of Wdpcp disrupts early chondrogenesis

**Fig. 4:**
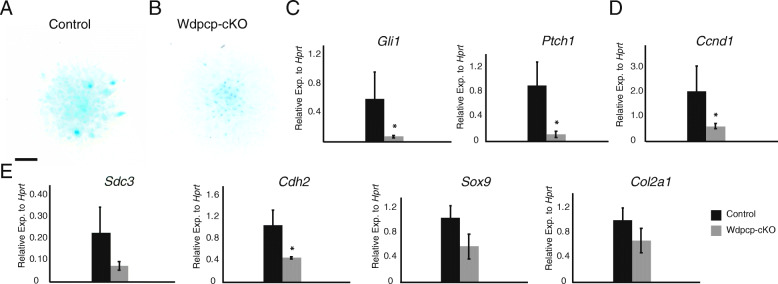
Loss of *Wdpcp* disrupts initiation of chondrogenesis. **A**, **B** Alcian Blue staining of day 3 E11.5 control and Wdpcp-cKO forelimb bud micromass cultures treated with hedgehog stimulating chondrogenic differentiation medium. **C** Expression of hedgehog reporter genes *Gli1* and *Ptch1* in control versus Wdpcp-cKO forelimb bud micromass cultures. **D** Expression of proliferation reporter gene *Ccnd1* in day 3 forelimb bud micromass cultures. **E** Expression of early pre-chondrogenic cellular condensation marker N-cadherin (*Cdh2*), early chondrogenic marker syndecan 3 (*Sdc3*), and chondrogenic differentiation markers *Sox9* and *Col2a1* in control versus Wdpcp-cKO day 3 forelimb bud cultures. Scale bar represents 200 μm in (**A**, **B**). Error bars represent one standard deviation. Control versus Wdpcp-cKO expression values were compared using Student’s t-test with * for *p* < 0.05

We examined the effect of Wdpcp-cKO on mesenchymal chondrogenic differentiation using high density micromass cultures of limb bud mesenchyme and treatment with SAG. We noted similar Alcian Blue staining between Wdpcp-cKO and control (Fig. 4A-B). Similar to results with mesenchymal progenitor cells, we observed higher expression of hedgehog reporter genes *Gli1* and *Ptch1* (Fig. 4C, D). Prior studies have shown that *Ccnd1* expression is closely correlated with proliferation in mesenchymal progenitors [36]. We found that increased expression of *Gli1 and Ptch1* corresponded with increased expression of the proliferation marker *Ccnd1* (Fig. 4D). *Sdc3* (Sydecan 3) (Fig. 4F) and *Cdh2* (N-Cadherin) are two markers of early chondrogenesis [37], and *Cdh2* upregulation is a robust marker of early chondrogenic differentiation [38]. We noted significantly decreased expression of *Cdh2* in Wdpcp-cKO versus control, suggesting reduced pre-chondrogenic cellular condensation (Fig. 4E). Early chondrogenic differentiation marker *Sdc3* as well as later chondrogenic differentiation markers *Sox9* and *Col2a1* (Fig. 4E) had lower expression in the Wdpcp-cKO versus control, but did not reach significance.

### Mesenchymal progenitor cells lacking Wdpcp have impaired hedgehog mediated osteogenic differentiation

**Fig. 5:**
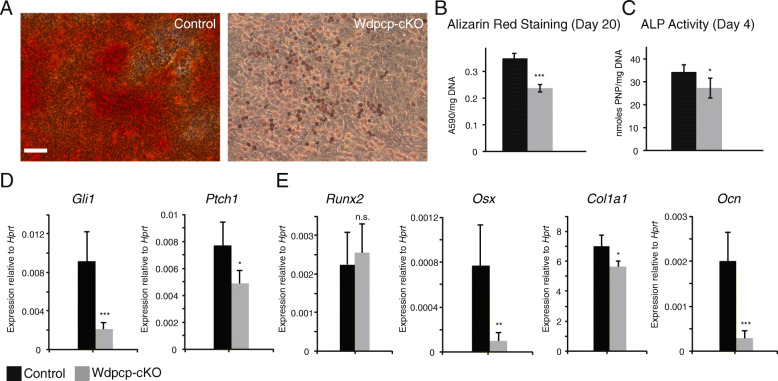
Loss of *Wdpcp* disrupts osteogenic differentiation. **A**, **B** Alizarin Red staining for mineralized matrix of control versus Wdpcp-cKO mesenchymal progenitor cells cultured in osteogenic medium for 20 days. **C** Alkaline phosphatase (ALP) activity in control versus Wdpcp-cKO mesenchymal progenitor cells cultured in osteogenic medium for 4 days. **D** Hedgehog pathway activity as assayed by expression of hedgehog reporter genes *Gli1* and *Ptch1* of control versus Wdpcp-cKO mesenchymal progenitor cells cultured in osteogenic medium for 20 days. **E** Expression of early osteogenic differentiation marker *Runx2* and mature osteogenic markers *Osx*, *Col1a1*, and *Ocn* in control versus Wdpcp-cKO mesenchymal progenitor cells cultured in osteogenic medium for 20 days.. Error bars represent one standard deviation. Effect of drug treatment within a genotype is analyzed using Student’s t-test with * for *p* < 0.05, ** for *p* < 0.01, *** for *p* < 0.001

Given the observation of delayed calcification (Fig. 2A, B) and expression of chondrocyte hypertrophy markers at the primary center of ossification (Fig. 2C, D), we wanted to investigate the effect of loss of Wdpcp in mesenchymal progenitor differentiation into osteoblasts. Mesenchymal progenitor cells were maintained in monolayer culture in osteogenic medium and treated with SAG. Alizarin Red S staining at 20 days of culture showed a dramatic reduction of mineralization in Wdpcp-cKO cells compared to control (Fig. 5A, B). Alkaline phosphatase activity at 4 days of culture showed significantly decreased activity in Wdpcp-cKO cells (Fig. 5C). Expression of hedgehog activity reporter genes *Gli1* and *Ptch1* at 20 days of culture was significantly decreased in Wdpcp-cKO (Fig. 5D). We quantified the expression of several markers of chondrocyte hypertrophy/osteoblast differentiation. *Runx2* is one of the key chondrocyte hypertrophy markers [39]. Expression of *Runx2* is necessary for expression of the key osteoblast transcription factor *Osx (Sp7*) [40]. *Ocn* and *Col1a1* are key components of osteoid matrix and characteristic markers of mature osteoblasts [28]. Expression of *Runx2* was not significantly different from control in Wdpcp-cKO (Fig. 5E). On the other hand, expression of *Sp7*, *Col1a1,* and *Ocn* (*Bglap2)* is significantly decreased in Wdpcp-cKO versus control (Fig. 5E). The lack of a significant difference in *Runx2* expression is likely due to its activity as an early transcriptional regulator of osteogenic differentiation, i.e., at an earlier time point than was measured in the osteogenesis assay relative to the expression of *Osx (Sp7)*, which depends on prior upregulation of *Runx2*.

## Discussion

The skeletal phenotype of the Wdpcp-cKO mice is similar to that of several other mutants with deficient ciliogenesis. Although mutations in proteins necessary for normal ciliogenesis typically result in perinatal lethality, some mouse models survive to later stages of development. A hypomorphic allele of the intraflagellar transport protein *Ift88*, the Tg737^orpk^ mouse, has shortened long bones and abnormal growth plate morphology with delayed expression of chondrocyte hypertrophy marker *Col10a1* [7]. Similarly, a hypomorphic allele of *Intu* demonstrates shortened long bones, delayed ossification, and delayed expression of hypertrophic markers with decreased hedgehog activation [20]. Mice with a hypomorphic mutation in *Poc1a*, which encodes a centriolar protein necessary for ciliogenesis, also display shortened long bones and disorganized growth plates [41]. Mice with an inactivating point mutation in *Ick* demonstrate shortened long bones and polydactyly with delayed hypertrophy and loss of hedgehog activation in the growth plate [42]. *Talpid3*^*−/−*^ mice survive to birth and have disrupted ciliogenesis and hedgehog signaling [43] with associated polydactyly, shortened long bones, and delayed chondrocyte hypertrophy with expanded zone of hypertrophy in the growth plate [44]. *Evc*^*−/−*^ and *Evc2*^*−/−*^ mice survive to birth and display no polydactyly, but do have shortened long bones. Evc localizes to the basal body of the chondrocyte primary cilium, and *Evc*^*−/−*^ mice display disorganized growth plate structure with decreased *Gli1* and *Ptch1* expression [26]. Unlike the *Wdpcp*^*Cys40/Cys40*^ mutant [17], *Evc*^*−/−*^ fibroblasts do not have a dramatic impairment in Gli3 full length to repressor processing, but do have decreased Gli1 expression in response to SAG [45]. Common to all of these models is a failure to activate hedgehog signaling and shortened appendicular skeletal elements.

The Wdpcp-cKO skeletal phenotype also has similarities with conditional knockout models of cilia related proteins. Prx1-Cre mice with conditional limb bud mesenchyme deletion of intraflagellar transport proteins [12, 46], kinesins [47], or basal body proteins [25] display polydactyly and shortened long bones with delayed ossification that correlates with disrupted cilia formation and loss of *Gli1* expression and hedgehog signaling abnormalities similar to the Wdpcp-cKO mouse. The Wdpcp-cKO mouse represents the first conditional limb bud mesenchyme knockout of a CPLANE protein. Col2a-Cre mice with conditional deletion of *Kif3a* or *Ift88* at a later time point in skeletal development after chondrogenesis do not have polydactyly and have mildly shortened long bones, but do have abnormal growth plates with decreased growth plate proliferation and decreased hedgehog activation similar to the Wdpcp-cKO mice [6]. These studies suggest that proliferation and growth prior to onset of *Col2a1* expression is an important contributor to the skeletal phenotype of the Wdpcp-cKO mice and other cilia deficient mice.

Mesenchymal proliferation and cellular condensation is essential for initiation of chondrogenesis to achieve the cell density required [34]. Additionally, tight regulation of hedgehog signaling is also critical for early initiation of chondrogenesis [29]. Recent studies of limb bud development have implicated hedgehog signaling as a key regulator of both mesenchymal limb bud progenitor proliferation and transition to chondrogenesis [36], and have highlighted the sensitivity of this process to small perturbations in the level of cilia associated hedgehog signaling activation [31]. Our findings of disrupted hedgehog signaling, decreased proliferative response, and decreased expression of early cellular condensation and chondrogenic markers in the Wdpcp-cKO mesenchymal progenitor cells is in line with these prior findings. Furthermore, they suggest that a significant contributing driver of the phenotype observed in the Wdpcp-cKO mice is related to perturbed hedgehog signaling in the later stages of limb bud mesenchyme proliferation and differentiation to early stages of chondrogenesis.

Hedgehog signaling represents the molecular pathogenic link between ciliary dysfunction and skeletal phenotype [48, 49]. Cilia coordinate the processing of the Gli transcription factors to their shortened repressor forms [31] as well as their fully activated forms downstream of hedgehog [50]. Similar to the Wdpcp-cKO mice, *Ihh*^*−/−*^ mice have shortened long bones, and knockdown of *Ihh* induces inhibition of cell proliferation and mature chondrocyte markers [51]. Prrx1-CreERT deletion of *Ptch1* in postnatal mice activates hedgehog signaling and promotes mesenchymal progenitor proliferation as well as differentiation into chondrocytes and osteoblasts [52]. Conversely, failure to activate hedgehog signaling in Wdpcp-cKO mesenchymal progenitors slows proliferation and differentiation into chondrocyte and osteoblast. Similarly, knockdown of *Ift80* and *Ift88* in cultured mesenchymal progenitors disrupts ciliogenesis and has been shown to decrease chondrocyte and osteoblast differentiation [8–10, 53].

Previous studies of *Wdpcp* null mice have demonstrated perturbed Wnt signaling in developing heart and PCP defects including disrupted cochlear hair cell alignment with loss of *Vangl2* expression as well as abnormal cell migration and cytoskeleton organization [17]. In the developing limb however, the Wdpcp-cKO mouse does not display a loss of flattening or columnar organization observed in other mutants of the PCP pathway [54–56]. Thus, while PCP may contribute in part to the observed skeletal phenotype of the Wdpcp-cKO mouse, the overlap in hedgehog signaling changes and phenotype with other cilia mutants suggests that these are significant components of the pathology. Additionally, prior studies have demonstrated that while Wnt signaling is perturbed in cilia deficient mutants, this is a downstream effect of the alteration of hedgehog signaling [57]. Interestingly, mice bearing a hypomorphic allele of the Wnt PCP receptor *Prickle1* have been found to have defective ciliogenesis [58, 59], underscoring the close interplay between cilia and PCP signaling. Future studies with the Wdpcp-cKO mouse will help to elucidate the specific roles of cilia and PCP in the developing limb and to refine our molecular model of limb development and growth plate organization.

## Conclusions

The generation of the Wdpcp-cKO mouse characterized in this study established the CPLANE protein Wdpcp as essential for mouse appendicular skeletal development in both the limb bud as well later stages of skeletal development including growth plate organization. We demonstrate that there is a significant impairment in the activation of hedgehog signaling that is associated with compromised proliferation and differentiation of skeletal tissues.

## Methods

### Mouse strains

The *Wdpcp*^*Cys40/Cys40*^ mouse and *Wdpcp*^*Flox/Flox*^ mouse have been previously described [17]. The *Prx1-Cre* mouse strain (Jackson Stock #005584) and *Gli1-Lacz* mouse strain (Jackson Stock #008211) were obtained from Jackson Laboratories. To avoid recombination in the female germline*,* male *Prx1-Cre;Wdpcp*^*Cys40/+*^ mice were crossed with the female *Wdpcp*^*Flox/Flox*^ mice to induce limb bud mesenchyme specific deletion of the *Wdpcp*^*Flox*^ allele [27], .resulting in litters with *Prx1-cre;Wdpcp*^*Flox/+*^ (control) and *Prx1-cre;Wdpcp*^*Flox/Cys40*^ (Wdpcp-cKO) mice. Male *Gli1-Lacz;Prx1-cre;Wdpcp*^*Cys40/+*^ and female *Wdpcp*^*Flox/Flox*^ mice were crossed for limb bud assay. Plug date was identified as E0.5 for timed matings. All reported mice are from mixed background strains. Mutant mice were obtained in the expected Mendelian ratio for each mating, and no association of phenotype was noted with sex. All histological specimens are shown in comparison to littermates and representative of at least 3 biological samples. Mice were under care of a veterinarian, and all mice used in this study were euthanized with carbon dioxide in a 10 L volume chamber with a flow rate of carbon dioxide at 5 L/min with flow maintained for 2 min following cessation of respiration followed by cervical dislocation in accordance with National Institutes of Health Office of Animal Care and Use guidelines. The study was carried out in compliance with ARRIVE guidelines.

### Genotyping

For genotyping, DNA was extracted from livers of embryos and tail clips of newly weaned mice using the REDExtract-N-Amp Tissue PCR Kit (Sigma). Primers used for genotyping are included in Table 1. The *Wdpcp*^*Cys40*^ allele was genotyped using Sanger sequencing of a 150 base pair fragment to identify the relevant A > G base pair change (Supplemental Fig. 1A). The *Wdpcp*^*Flox*^ allele was genotyped using polymerase chain reaction (PCR) with primers to amplify a region containing an inserted flox site (424 base pair fragment) or lacking an inserted flox site (262 base pair fragment) that could be differentiated with agarose gel electrophoresis [17]. Presence of the *Prx1-Cre* allele was identified by PCR with primers specific to *Cre* that amplified a 102 base pair fragment (Supplemental Fig. 1B,C) [60].

**Table 1:** Genotyping primers

Primer Name	Target Allele	Sequence (5′-3″)
Cys40-F	*Wdpcp*^*Cys40*^	CATGTTTTATTTGCCAGCACGAG
Cys40-R	*Wdpcp*^*Cys40*^	GCGAGAGCCAGTCCTCTATG
Flox-F	*Wdpcp*^*Flox*^	GGTTTCAAAAATGGGAGCAA
Flox-R	*Wdpcp*^*Flox*^	CTGCTTTGCATCAGTTCCTG
Cre-F	Prx1-Cre	ACTTGGCAGCTGTCTCCAAG
Cre-R	Prx1-Cre	GCGAACATCTTCAGGTTCTG

### Skeletal preparations

Skeletal preparations were made by co-staining embryos with Alizarin Red S for ossified, calcium rich tissue and Alcian Blue for cartilage as described previously [61]. Briefly, embryos were scalded in hot tap water, skinned, and their abdominal and thoracic organs removed. Livers were saved for genotyping. Embryos were transferred into ethanol before staining 24 h with 40% glacial acetic acid, 60% ethanol, 0.0001% Alcian Blue (Sigma) solution at room temperature. Embryos were destained for 24 h in ethanol before being transferred to a solution containing 2% KOH (Sigma) in distilled water with 0.0015% Alizarin Red S (Sigma) for 5 h. Embryos were subsequently destained in 2%KOH in distilled water overnight, followed by 1% KOH in 50% glycerol/50% distilled water. Embryos were transferred to 50% glycerol/50% distilled water for imaging. All images are representative of at least 3 specimens. For limb length quantification, the longest vector along each cartilage element was used. Six limbs from six separate animals were used for both mutants and controls.

### Quantitative real time RT-PCR (qRT-PCR)

Total RNA was extracted using TRIzol (Invitrogen) with RNeasy columns (Qiagen) for purification and removal of genomic DNA. RNA was quantified on nanodrop, and cDNA was synthesized from 200 ng of total RNA using Superscript III (Invitrogen) with the Oligo (dT) primers per the manufacturer’s protocol. Quantitative real-time PCR was performed on Applied BioSystems StepOnePlus with Applied Biosystems SYBR Green PCR mastermix in 96-well plates. Primers were all used at a concentration of 200 nM. Cycling variables were as follows: 95 **°**C for 10 min, then 40 cycles of 15 s denaturation at 95 **°**C and 1 min at 60 **°**C. Primer sequences are included in Table 2. Expression was reported normalized to housekeeping gene *HPRT* due to reported stability of expression of this gene during skeletal development [62, 63]. The mean and standard deviation represent 3–4 biological replicates each composed of RNA isolated from 3 experimental replicates. All statistics were performed on difference from *HPRT* cycle counts to avoid error propagation.

**Table 2:** qRT-PCR primers

Gene	P	Reference	Sequence (5′-3′)	Accession No.
Hprt	5’	MGH PrimerBank	TCAGTCAACGGGGGACATAAA	NM_013556
Hprt	3’	MGH PrimerBank	GGGGCTGTACTGCTTAACCAG	NM_013556
Gli1	5’	MGH PrimerBank	CCAAGCCAACTTTATGTCAGGG	NM_010296
Gli1	3’	MGH PrimerBank	AGCCCGCTTCTTTGTTAATTTGA	NM_010296
Ptch1	5’	MGH PrimerBank	GCCTTCGCTGTGGGATTAAAG	NM_008957
Ptch1	3’	MGH PrimerBank	CTTCTCCTATCTTCTGACGGGT	NM_008957
Wdpcp	5’	MGH PrimerBank	GCTTGACTGAACTACACCTGTG	NM_145425
Wdpcp	3’	MGH PrimerBank	TGAGTGTCCAAGGATAATCTCGT	NM_145425
Ccnd1	5’	Lopez rios 2012	CAGACGTTCAGAACCAGATTC	NM_007631
Ccnd1	3’	Lopez rios 2012	CCCTCCAATAGCAGCGAAAAC	NM_007631
Sdc3	5’	MGH PrimerBank	AGAGGCCGGTGGATCTTGA	NM_011520
Sdc3	3’	MGH PrimerBank	CTCCTGCTCGAAGTAGCCAGA	NM_011520
Cdh2	5’	MGH PrimerBank	AGCGCAGTCTTACCGAAGG	NM_007664
Cdh2	3’	MGH PrimerBank	TCGCTGCTTTCATACTGAACTTT	NM_007664
Col2a1	5’	MGH PrimerBank	GGGAATGTCCTCTGCGATGAC	NM_031163
Col2a1	3’	MGH PrimerBank	GAAGGGGATCTCGGGGTTG	NM_031163
Sox9	5’	MGH PrimerBank	AGTACCCGCATCTGCACAAC	NM_011448
Sox9	3’	MGH PrimerBank	ACGAAGGGTCTCTTCTCGCT	NM_011448
Runx2	5’	MGH PrimerBank	CCGCCTCAGTGATTTAGGGC	NM_009820
Runx2	3’	MGH PrimerBank	GGGTCTGTAATCTGACTCTGTCC	NM_009820
Sp7	5’	MGH PrimerBank	ATGGCGTCCTCTCTGCTTG	NM_130458
Sp7	3’	MGH PrimerBank	TGAAAGGTCAGCGTATGGCTT	NM_130458
Bglap2	5’	MGH PrimerBank	CTGACCTCACAGATCCCAAGC	NM_001032298
Bglap2	3’	MGH PrimerBank	TGGTCTGATAGCTCGTCACAAG	NM_001032298
Col1a1	5’	MGH PrimerBank	TAAGGGTCCCCAATGGTGAGA	NM_007742
Col1a1	3’	MGH PrimerBank	GGGTCCCTCGACTCCTACAT	NM_007742

### Western blotting

The limb bud specific knockdown of Wdpcp was confirmed by individually genotyping embryos after forelimb buds had harvested for protein isolation. Forelimb bud protein content was isolated with Total Protein Extraction Kit (Millipore) supplemented with 5 mM EDTA and 1X Halt Protease and Phosphatase Inhibitor Cocktail (Thermo Scientific). Samples were sonicated to homogenize lysate and protein concentration was quantified using BCA assay kit (Pierce) and standardized to same concentration using isolation buffer dilution. Western blots were performed as previously described [64]. Protein samples were subjected to reducing SDS-PAGE and transferred to low-fluorescence background polyvinyl fluoride (PVDF) membranes (Millipore). Membranes were blocked in 3% milk in 0.25% Tween-20 in TBS (TBS-T) for 1 h at room temperature and probed overnight at 4C with primary antibody in 1% milk/TBS-T. Primary antibodies used were goat anti-Wdpcp (1:1000) (Santa Cruz, Sc-245,737 T-20) and mouse anti-β-actin (Santa Cruz sc-47,778) (1:1000). After washing with TBS-T, membranes were incubated for 1 h at room temperature with HRP conjugated anti-mouse (Abcam) (1:1000) or poly-HRP conjugated anti-goat antibody (Pierce) (1:2000) in 1% milk/TBS-T. Immunoreactive bands were visualized with SuperSignal West Dura Extended Duration Substrate (Thermo Scientific) on a Fotodyne imaging system. Each blot was repeated in duplicate, and representative blot is presented.

### Hedgehog responsiveness experiments

Mesenchymal progenitor cells used in these experiments were isolated and expanded from E13.5 forelimbs. For all drug treatment experiments, cells were grown to confluence, trypsinized, and re-plated at full confluence (150,000 cells/cm^2^) in appropriate size culture well in growth medium for 1 day before changing to starvation medium (DMEM supplemented with 0.25% MSC qualified FBS). Previous studies had demonstrated that cell cycle arrest as induced by serum starvation and confluence is necessary to induce optimal ciliation and hedgehog responsiveness [65]. Following 2 days of culture with starvation medium, medium was changed to starvation medium supplemented with pharmacological agent dissolved in DMSO (Sigma) for 24 h. RNA for RT-PCR and/or protein for Western Blots was isolated from cells at this time point. For expression studies, results presented are from 3 independent biological replicates. All drugs were diluted in dimethyl sulfoxide (DMSO) and added 1:1000 to culture medium. DMSO was added 1:1000 for carrier control. The Click-iT Plus Edu 594 assay was used to quantify cell proliferation over final 24 h of cell culture. Following serum starvation period, 10 mM EdU dissolved in PBS was added to culture medium at 1:1000 for a final concentration of 10 uM. Following fixation with 4% paraformaldehyde for 15 min at room temperature, EdU staining was performed per the manufacturer’s protocol (Lifetechnologies). Hoechst staining was performed to identify all nuclei. Images were acquired on Zeiss inverted epifluorescent microscope with 10X objective. Three fields were captured for each treatment condition and presented images are representative. Three biologic replicates were performed per experimental condition.

### Micromass culture

Forelimbs were obtained from 11.5 dpc *Prx1-cre;Wdpcp*^*Cys40/Flox*^ and control embryos. Micromass cultures were prepared as previously described [66–68]. Dissected limbs were dissociated in 1 unit/mL dispase (Stem Cell Technologies) for 1.5 h at 37 °C. Digested limbs were pipetted up and down with 200 uL pipette to break up cells before being diluted in 10 mL 2:3 DMEM/F-12 medium containing 10% MSC qualified FBS (Invitrogen) to neutralize dispase. This suspension was passed through a 40 um filter and spun down to a pellet at 1200 g. Cells were resuspended in 2:3 DMEM/F-12 medium and counted with hematocytometer. Cells were then divided into experimental groups, spun down and resuspended in 2:3 DMEM/F-12 medium supplemented with either DMSO or appropriate pharmacological agent dissolved in DMSO. Cells were diluted to 10 million cell/mL and spotted in 10 uL droplets on Nunc 24–well culture dishes. Cells were allowed to adhere for 1.5 h in a cell incubator with humidified atmosphere containing 5% CO_2_. After 2 h, wells were flood with growth medium containing appropriate pharmacological agent. Cultures were harvested after 3 days. For Alcian Blue staining, cultures were fixed for 15 min at room temperature in 4% paraformaldehyde in 1X phosphate buffered saline before staining overnight at 4 °C with 1% Alcian Blue stain solution pH 1.0 (EK Industries). Stained cultures were washed 3x with phosphate buffered saline before imaging. Alcian Blue staining shows a representative image of 3 to 4 independent biological replicates [60].

### Histology and immunohistochemistry

Specimens were fixed in 4% paraformaldehyde at indicated time point for 24 h overnight at 4C before being dehydrated and paraffin embedded. Forelimbs were sectioned at 7 μm thickness. For Safranin O/Fast Green staining, sections were deparaffinized and rehydrated before being stained in Weigert’s Iron Hematoxylin (Sigma) and 0.02% aqueous Fast Green (Sigma) followed by a rinse in 1% acetic acid and 0.1% aqueous Safranin-O (Sigma). For immunohistochemistry, ImmPRESS Excel amplified polymer staining kit (Vector Labs) was used [60]. Primary antibodies used for immunohistochemistry were rabbit anti-Osx/Sp7 (1:500) (Abcam, Ab22552), rabbit anti-Runx2 (1:500) (Santa Cruz, Sc-10,758 M-70), and rabbit anti-Gli1 (Santa Cruz, Sc-20,687 H-300) (1:200). For immunocytochemistry, mesenchymal progenitors were plated on chamber slides and allowed to adhere for 24 h. Cells were then fixed with 4% paraformaldehyde and stained with mouse anti-acetylated alpha tubulin (1:200) (R&D systems T7451 6-11B-1) overnight at 4C followed by chicken anti-mouse Alexafluor 647 (1:200) (Invitrogen) and DAPI counterstain.

### Mesenchymal progenitor isolation

Mesenchymal progenitor cells were isolated from the limbs of E13.5 embryos that were digested in 0.5% Trypsin EDTA and mechanically disrupted. Trypsin was neutralized after 5 min at 37C with DMEM supplemented with 10% MSC qualified FBS (Gibco) with 1X Penicillin/Streptomycin/Fungizone (Gibco) growth medium. Cells were pipetted up and down to break up remaining tissue before spinning at 1000 g to pellet in 15 mL Vulcan tube. Cells were then resuspended in growth medium and plated on T75 at what was considered passage 0. Cells were expanded 2 days with growth medium changed each day. Cells were passaged on day 2 and split (1:4). Cells were grown to 90% confluence, passaged, and frozen in cryoprotective freezing medium (Lonza) for storage in liquid nitrogen. All experiments using were conducted at passage 3 or 4 [60].

### Osteogenic differentiation assays

Following expansion, mesenchymal progenitor cells were seeded at 70% confluence (100,000 cells/cm^2^) in growth medium for 2 days before changing to standard osteogenic medium containing 1 mM β-glycerolphosphate, 100 nM ascorbic acid, and 20 ng/mL rBMP2 (Peprotech) supplemented with 10 nM smooth agonist (SAG, Calbiochem) similar to prior studies [10, 53]. Osteogenic medium was changed every 4 days. Alkaline phosphatase activity was measured with paranitrophenyl phosphate assay (Sigma) at day 4 of osteogenic differentiation. Eight independent biological replicates were used for quantification. Alizarin red staining was used to assay calcium matrix deposition of osteogenic cultures. Cells were fixed in 70% ethanol before being stained for 15 min with 2% alizarin red solution at pH 4.2 (Rowley Biochemical Institute). For quantification, 1 mL of cetylpyridinium chloride was used per 6 well plate well to solubilize dye. DNA was isolated from an analogous 6 well plate well with 1 mL of 0.5% Triton X-100 in distilled water and quantified using Quant-iT PicoGreen dsDNA Assay Kit (Life Technologies). Soluble alizarin red was quantified spectroscopically (A_580_) [60]. Six independent biologic replicates were used for quantification.

## Supplementary Information

**Additional file 1: Figure S1.** Genotyping of Wdpcp^Cys40^, Wdpcp^Flox^, and Prx1-cre mice. The *Cys40* allele is a result of an A- > G base change at the end of exon 5 that causes a splice defect with exon 5 excluded from the mRNA. This splice defect results in a premature stop codon (Cui et al. 2013). This allele is genotyped by Sanger sequencing (A). The construct for the inducible Wdpcp deletion had a PGKNeo cassette flanked by FRT sites that was removed via breeding to a mouse with constitutive expression of the FLP recombinase. The conditional mouse model used in this study carries two LoxP sites on either side of exon 5 (Cui et al. 2013). Cre mediated recombination results in deletion of exon 5, forming a functional null that mimics the *Cys40* mutant. Mice carrying the Prx1-cre allele were identified by PCR amplification of a region of the Cre recombinase cDNA (B) that resulted in a 102 bp product (C). Mice carrying the floxed allele of *Wdpcp* were identified with primers that amplified a fragment containing (424 bp) or lacking (262 bp) the inserted LoxP site (C). **Figure S2.** Defective ciliogenesis in Wdpcp-cKO mesenchymal progenitor cells. Mesenchymal progenitors from control and Wdpcp-cKO mice were stained for acetylated alpha tubulin (red), a marker of primary cilia (white arrows). Acetylated alpha tubulin is a modified form of alpha tubulin found in high concentrations within primary cilia. Nuclei were stained with DAPI (blue) for counterstain.

## Data Availability

All data generated or analysed during this study are included in this published article.
