# Supplementary material for: Wdpcp regulates cellular proliferation and differentiation in the developing limb via hedgehog signaling
Source: BMC Dev Biol. 2021 Jul 5;21:10. doi: 10.1186/s12861-021-00241-9 (PMC8258940; doi:10.1186/s12861-021-00241-9)
Supplement: Supplementary file 1 — Additional file 1: Figure S1. Genotyping of WdpcpCys40, WdpcpFlox, and Prx1-cre mice. The Cys40 allele is a result of an A- > G base change at the end of exon 5 that causes a splice defect with exon 5 excluded from the mRNA. This splice defect results in a premature stop codon (Cui et al. 2013). This allele is genotyped by Sanger sequencing (A). The construct for the inducible Wdpcp deletion had a PGKNeo cassette flanked by FRT sites that was removed via breeding to a mouse with constitutive expression of the FLP recombinase. The conditional mouse model used in this study carries two LoxP sites on either side of exon 5 (Cui et al. 2013). Cre mediated recombination results in deletion of exon 5, forming a functional null that mimics the Cys40 mutant. Mice carrying the Prx1-cre allele were identified by PCR amplification of a region of the Cre recombinase cDNA (B) that resulted in a 102 bp product (C). Mice carrying the floxed allele of Wdpcp were identified with primers that amplified a fragment containing (424 bp) or lacking (262 bp) the inserted LoxP site (C). Figure S2. Defective ciliogenesis in Wdpcp-cKO mesenchymal progenitor cells. Mesenchymal progenitors from control and Wdpcp-cKO mice were stained for acetylated alpha tubulin (red), a marker of primary cilia (white arrows). Acetylated alpha tubulin is a modified form of alpha tubulin found in high concentrations within primary cilia. Nuclei were stained with DAPI (blue) for counterstain. [file 12861_2021_241_MOESM1_ESM.docx]

Title: Wdpcp regulates proliferation and differentiation in the developing limb via hedgehog signaling

Authors: Mark T. Langhans, Jingtao Gao, Ying Tang, Bing Wang, Peter Alexander, and Rocky S. Tuan


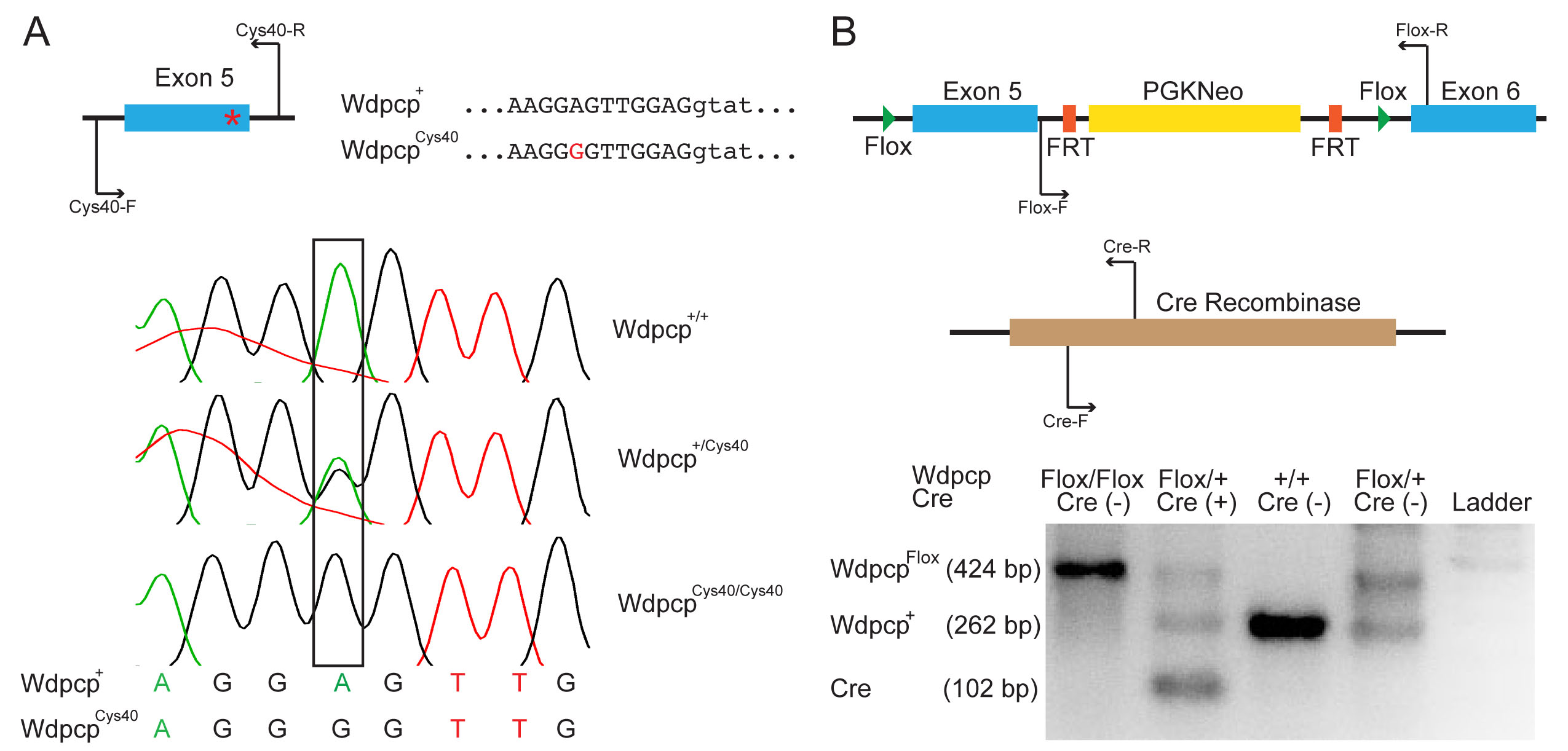


Figure S1. Genotyping of Wdpcp^Cys40^, Wdpcp^Flox^, and Prx1-cre mice. The *Cys40* allele is a result of an A->G base change at the end of exon 5 that causes a splice defect with exon 5 excluded from the mRNA. This splice defect results in a premature stop codon (Cui et al. 2013). This allele is genotyped by Sanger sequencing (A). The construct for the inducible Wdpcp deletion had a PGKNeo cassette flanked by FRT sites that was removed via breeding to a mouse with constitutive expression of the FLP recombinase. The conditional mouse model used in this study carries two LoxP sites on either side of exon 5 (Cui et al. 2013). Cre mediated recombination results in deletion of exon 5, forming a functional null that mimics the *Cys40* mutant. Mice carrying the Prx1-cre allele were identified by PCR amplification of a region of the Cre recombinase cDNA (B) that resulted in a 102 bp product (C). Mice carrying the floxed allele of *Wdpcp* were identified with primers that amplified a fragment containing (424 bp) or lacking (262 bp) the inserted LoxP site (C).

**Figure S2. Defective ciliogenesis in Wdpcp-cKO mesenchymal progenitor cells.** Mesenchymal progenitors from control and Wdpcp-cKO mice were stained for acetylated alpha tubulin (red), a marker of primary cilia (white arrows). Acetylated alpha tubulin is a modified form of alpha tubulin found in high concentrations within primary cilia. Nuclei were stained with DAPI (blue) for counterstain.


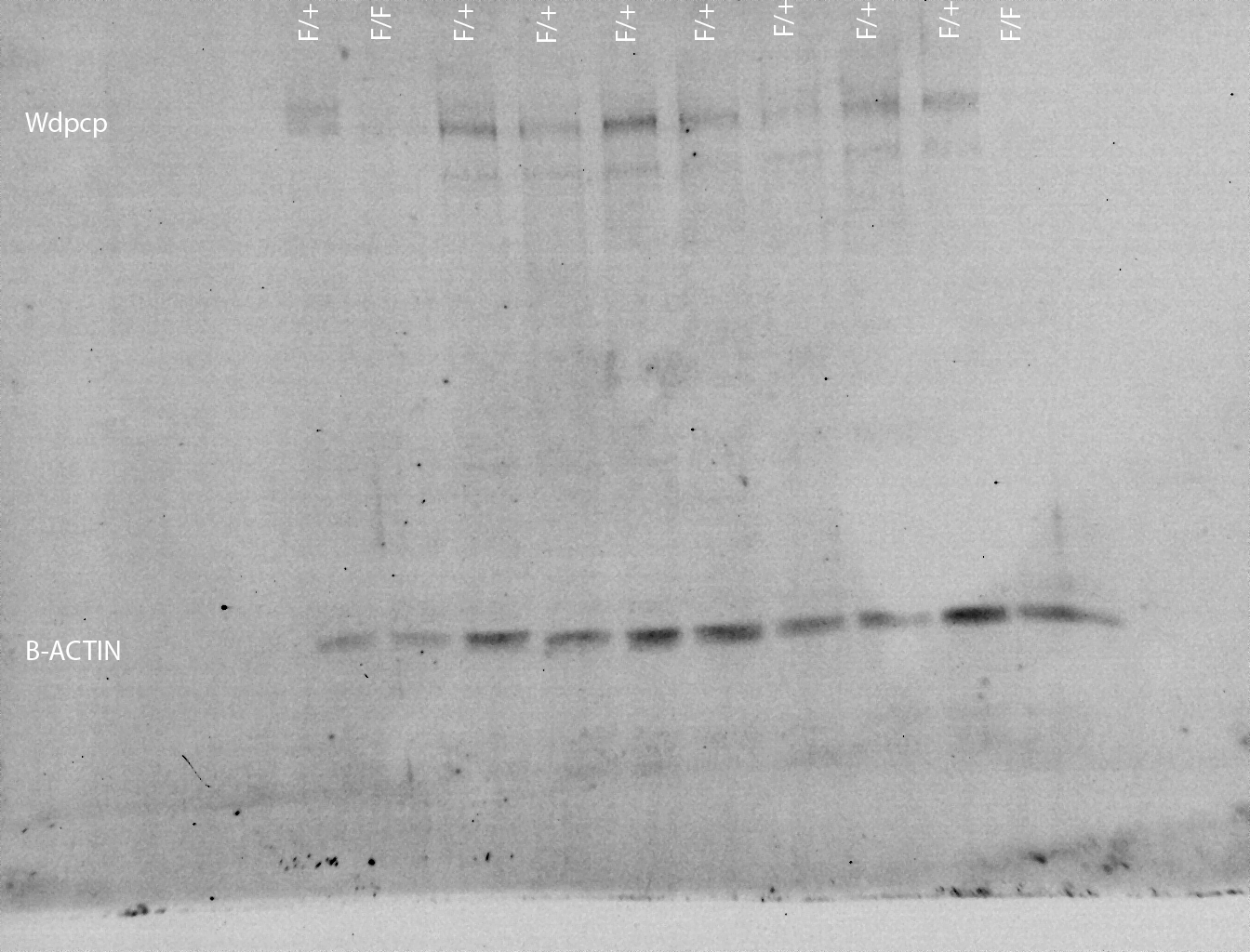


Figure 1 cropped Wdpcp/Bactin Gel
